# Conformational templating of α-synuclein aggregates in neuronal-glial cultures

**DOI:** 10.1186/1750-1326-8-17

**Published:** 2013-05-28

**Authors:** Amanda N Sacino, Michael A Thomas, Carolina Ceballos-Diaz, Pedro E Cruz, Awilda M Rosario, Jada Lewis, Benoit I Giasson, Todd E Golde

**Affiliations:** 1 Department of Neuroscience, McKnight Brain Institute, Center for Translational Research in Neurodegenerative Disease, University of Florida College of Medicine, 1275 Center Drive, Gainesville, FL 32610, USA

**Keywords:** α-Synuclein, Parkinson’s disease, Self-templating, Amyloid, Prion

## Abstract

**Background:**

Genetic studies have established a causative role for α-synuclein (αS) in Parkinson’s disease (PD), and the presence of αS aggregates in the form of Lewy body (LB) and Lewy neurite (LN) protein inclusions are defining pathological features of PD. Recent data has established that extracellular αS aggregates can induce intracellular αS pathologies supporting the hypothesis that αS pathology can spread via a “prion-like” self-templating mechanism.

**Results:**

Here we investigated the potential for conformational templating of αS intracellular aggregates by seeding using recombinant wild-type and PD-linked mutant (A53T and E46K) αS in primary mixed neuronal-glial cultures. We find that wild-type and A53T αS fibrils predominantly seed flame-like inclusions in both neurons and astrocytes of mixed primary cultures; whereas the structurally distinct E46K fibrils seed punctate, rounded inclusions. Notably, these differences in seeded inclusion formation in these cultures reflect differences in inclusion pathology seen in transgenic mice expressing the A53T or E46K αS mutants. We further show that the inclusion morphology is dictated primarily by the seed applied rather than the form of αS expressed. We also provide initial evidence that αS inclusion pathology can be passaged in primary astrocyte cultures.

**Conclusion:**

These studies establish for the first time that αS aggregation in cultured cells can occur by a morphological self-templating mechanism.

## Background

Parkinson’s disease (PD) is the most common movement neurodegenerative disorder [1]. PD belongs to a spectrum of neurodegenerative diseases, termed α-synucleinopathies, unified by the presence of the brain accumulation of neuronal and glial α-synuclein (αS) inclusions [2,3]. The physiologic function of αS is still not completely established; however, αS gene (*SNCA*) defects that cause autosomal-dominant PD (*SNCA* gene duplication/triplication and missense mutations A30P, E46K, G51D, H50Q, and A53T) have clearly established a causal role for αS in disease [4-13]. Differences in the clinical profiles and pathology of PD patients and mouse models with either the A53T or E46K mutation have been documented [4,5,14-16]. *In vitro* biochemical studies on amyloidogenic αS fibrils derived from A53T and E46K missense mutations have also shown differences in nucleation and elongation of fibril polymerization, peptide structural order, and ultrastructural morphology [17-21].

Recent postmortem studies in PD patients suggest that the spread of pathology can occur intercellularly [22-26], and the induction of αS pathology in experimental mouse models using intracerebral injection of recombinant αS fibrils suggests that amyloidogenic αS may also spread by a “prion-like” mechanism [27,28]. A key characteristic of “prion-like” transmission is permissive templating, in which the amyloidogenic form of the protein interacts with normal endogenous protein, and that interaction induces a conformational change in the endogenous protein to an amyloidogenic β-pleated sheet structure [29,30]. A major difference between “prion-like” transmission and classical prion disease is paucity of inter-organism transmission. For many amyloids this “prion-like” conversion of protein conformation can often have unique structural and morphological properties that can be transmitted and this phenomenon has been termed “strain-specific” [29,30].

Taking advantage of previous *in vitro* observations that wild-type, A53T, and E46K αS amyloids have distinct structural properties [17-21,31] and that pathological inclusions in A53T and E46K transgenic mice are also distinct [14,15], we used a novel recombinant adeno-associated virus (rAAV)-mediated αS primary neuronal-glial culture model to test the hypothesis that fibrillar A53T and E46K αS may seed cellular inclusions with unique morphological properties. We show that neuronal and astrocytic inclusions formed by fibrillar A53T and E46K αS are morphologically consistent with those in transgenic mice and that the seeding fibrils (i.e., A53T versus E46K αS) have a dominant effect over the αS protein expressed. We also find that inclusion pathology induced by exogenous fibrils in one astrocyte culture can be passaged to a second astrocyte culture. This data provides important support for a conformational templating of αS inclusion pathology.

## Results

### Induction of αS aggregates in primary mixed neuronal-glial cultures

**Figure 1:**
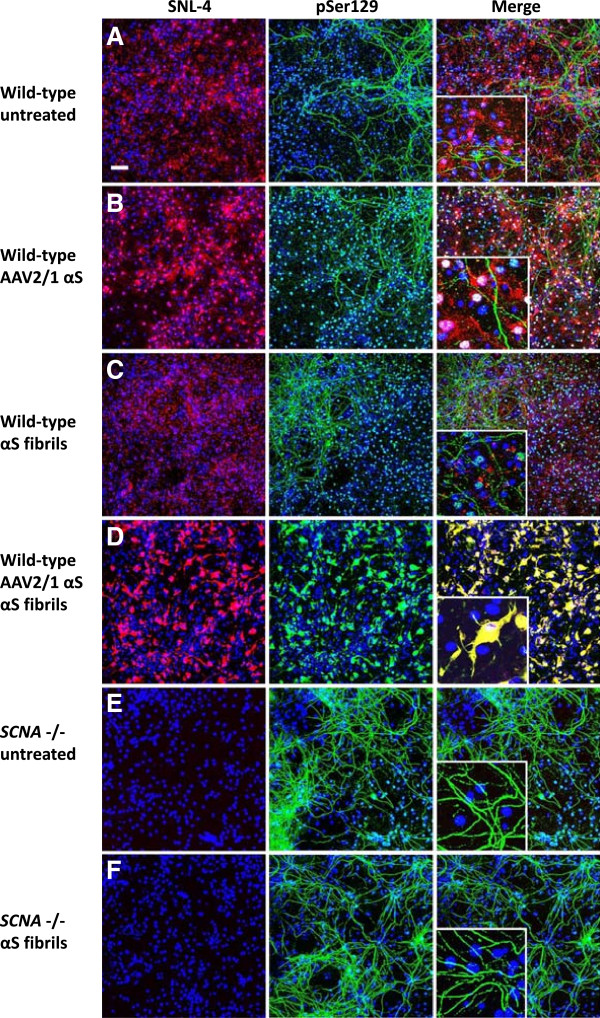
**αS seeded aggregate formation in rAAV2/1-mediated αS expression of mixed neuronal-glial cultures.** Double immunofluorescence with SNL-4 (red) and pSer129 (green): (**A**) In untreated cultures, αS demonstrated predominantly a punctate staining profile reflecting a presynaptic distribution, as previously demonstrated [39,40] and (**B**) rAAV2/1-mediated overexpression of αS increases both the presynaptic and nuclear staining intensity for αS. In these cultures, pSer129 demonstrates a neuritic staining profile that does not appear to be due to αS. (**C**) Addition of fibrils alone resulted in pSer129 staining of processes also seen in blank controls, but not overlapping with SNL-4. (**D**) Both rAAV2/1-mediated overexpression of αS and the addition of fibrils resulted in pSer129 immunoreactive inclusions in both the cell body and processes that overlaps with SNL-4. In cultures derived from *SNCA* null mice, both (**E**) untreated and (**F**) addition of fibrils resulted in a neuritic staining profile with pSer129, in the absence of SNL-4 staining. The addition of fibrils did not increase the amount of pSer129 neuritic staining. Cultures were counter stained with DAPI (blue). Bar scale = 100 μm; insets = 25 μm.

In previous studies, we and others [32-34] have shown that under experimental conditions that promote the entry of exogenous recombinant αS fibrils, the formation of robust Lewy body (LB)-like inclusions can be induced in cultured cell lines overexpressing human αS. To determine if we could induce inclusion formation in primary mixed mouse neuronal-glial cultures, we tested whether overexpression of wild-type human αS mediated by rAAV2/1 vectors and addition of amino-terminally truncated exogenous recombinant wild-type (21–140) αS fibrils was sufficient to induce αS aggregate formation. We assessed αS aggregate formation using several previously established methods. First, we used anti-pSer129 αS antibody immunoreactivity, since αS inclusions are hyperphosphorylated at Ser129 and this modification is an excellent marker of aggregate formation [32-37]. However, in primary neuronal cultures used here this antibody also recognizes a non-αS target localized only in MAP2 positive neuronal processes, as it stains these processes in cultures from wild-type and *SNCA* null mice (Figure 1). Therefore, co-localization of pSer129 and a second αS antibody, SNL-4, were used to track inclusion formation. Notably, the SNL-4 antibody binds the extreme amino terminus of αS. Using seeding with exogenous 21–140 and full-length αS fibrils, we previously showed that this antibody also stains the intracellular inclusions [32,38] and therefore can be used to distinguish the non-specific neuritic background staining seen with pSer129. In the primary mixed neuronal-glial cultures, αS predominantly demonstrated a punctate neuronal staining profile reflecting a presynaptic distribution (Figure 1A), as previously demonstrated [39,40]. rAAV2/1-mediated overexpression of αS increased both the presynaptic and nuclear staining intensity for αS, but did not affect the neuritic staining of the pSer129 non-αS target (Figure 1B). Addition of preformed fibrils alone resulted in pSer129 staining of processes similar to that seen in blank controls, but this staining did not co-localize with SNL-4 (Figure 1C). Addition of exogenous fibrils and overexpression of αS were both required to induce αS aggregate formation (Figure 1D). When robust aggregate formation is present, the staining of these aggregates with pSer129 is much more robust then the non-αS target, therefore the weaker signal of the non-αS target is more difficult to observe when capturing images that are appropriate to visualize pSer129 labeled αS aggregates.

In this system where we overexpressed αS using rAAV2/1 and added extracellular preformed αS fibrils to induce intracellular aggregate formation, inclusion formation was rapid and efficient. Using rAAV2-1 wild-type αS and wild-type αS fibrils, 0.4±0.1% and 29.1±3.8% of the transduced cells formed aggregates by 1 and 4 days following fibril addition, respectively. However, aggregate formation was accelerated if either E46K or A53T mutant αS was expressed or used for seeding as demonstrated by the percentage of transduced cells with aggregates at 1 day following the addition of fibrils: rAAV2-1 wild type-αS/A53T fibrils (1.5±0.2%), rAAV2-1 wild type-αS/E46K fibrils (2.6±1.2%), rAAV2-1 A53T αS/A53T fibrils (16.3±6.7%), rAAV2-1 A53T αS/E46K fibrils (7.9±2.4%), rAAV2-1 A53T αS/wild-type fibrils (11.9±1.9%), rAAV2-1 E46K αS/A53T fibrils (3.5±0.6%), rAAV2-1 E46K αS/E46K fibrils (1.7±0.3%), rAAV2-1 E46K αS/wild type fibrils (3.2±0.6%).

**Figure 2:**
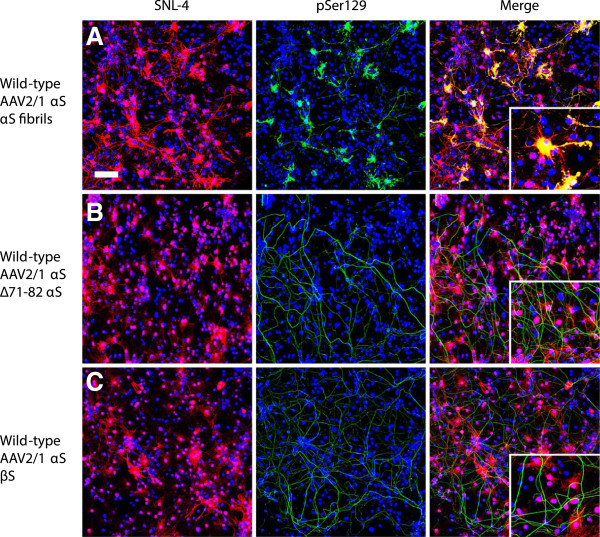
**Non-amyloidogenic synuclein proteins do not induce αS inclusion pathology.** Double immunofluorescence with SNL-4 (red) and pSer129 (green): Mixed neuronal-glial cultures with rAAV2/1-mediated overexpression of αS were treated with (**A**) fibrillar αS, (**B**) ∆71-82 αS or (**C**) β-synuclein. Only treatment with fibrillar αS resulted in Ser129 hyperphosphorylated αS aggregates. Cultures were counter stained with DAPI (blue). Bar scale = 100 μm; insets = 25 μm.

As additional controls to show that seeding with amyloidogenic αS was required for intracellular aggregate formation, we treated cultures overexpressing αS with β-synuclein or non-amyloidogenic ∆71-82 αS (Figure 2) [33,41]. Treatment with either of these proteins did not result in intracellular aggregate formation, but non-specific pSer129 staining in neuronal processes was still apparent.

**Figure 3:**
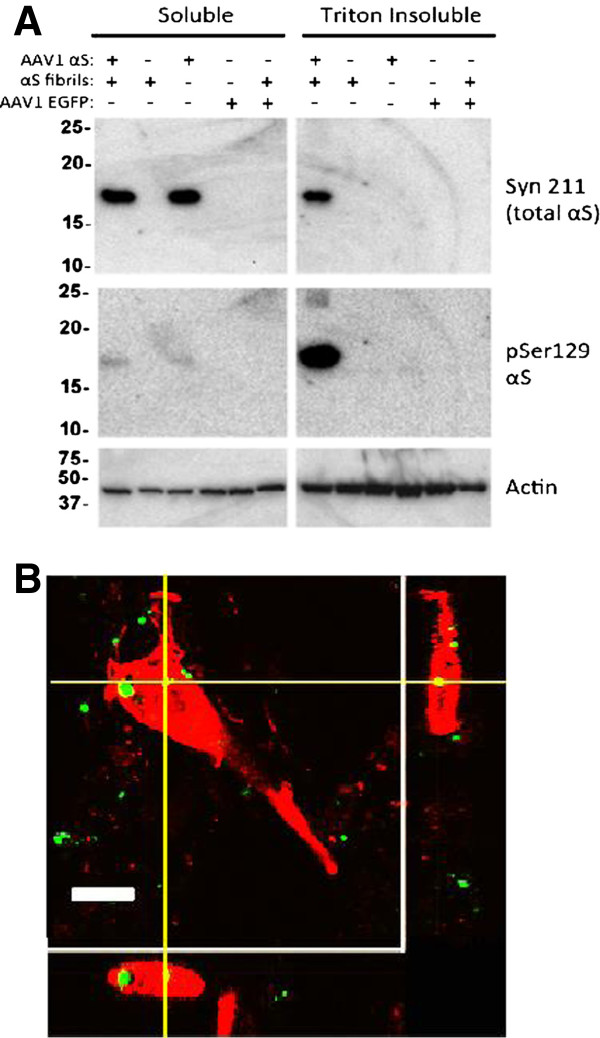
**Extracellular αS fibril uptake by cells and formation of intracellular Triton-insoluble αS aggregates.** (**A**) Formation of intracellular Triton-insoluble αS aggregates induced by extracellular αS amyloid fibrils. Cultures were treated with either rAAV2/1 wild-type αS or rAAV2/1 EGFP (green fluorescent protein control) alone or in combination with wild-type αS fibrils. Four days post-seeding, cultures were biochemically fractionated using Triton X-100 containing buffer as described in Materials and Methods. Aggregated αS is found in the Triton-insoluble fraction. Syn211, which recognizes the overexpressed human αS, showed bands in the soluble fractions treated with rAAV2/1 wild-type αS. Only with both the rAAV2/1-mediated overexpression of human αS plus the addition of extracellular αS fibrils is αS present in the Triton-insoluble fraction. pSer129, which recognizes the hyperphosphorylated aggregates, also showed a band for αS with both the overexpression of αS plus αS fibrils in the Triton-insoluble fraction. (**B**) Z-slice section analysis of αS inclusions showing incorporation of extracellular fibrils into aggregates. Thioflavin-S pre-labeled wild-type αS fibrils (green) are incorporated into pSer129 aggregates (red). Bar scale = 10 μm.

To establish that these pSer129 and SNL-4 immunostained aggregates were bonafide inclusions, we conducted biochemical fractionation and Western blot analysis previously used to validate the presence of Triton-insoluble αS aggregates in culture [32]. As shown in Figure 3A, rAAV-mediated αS overexpression in combination with the addition of extracellular αS fibrils induced the formation of Ser129 hyper phosphorylated Triton-insoluble aggregates.

To validate that extracellular, exogenous aggregates could enter cells and be incorporated into intracellular αS inclusions, Thioflavin-S pre-labeled recombinant αS fibrils were added to cultures and shown to be present within intracellular aggregates (Figure 3B).

**Figure 4:**
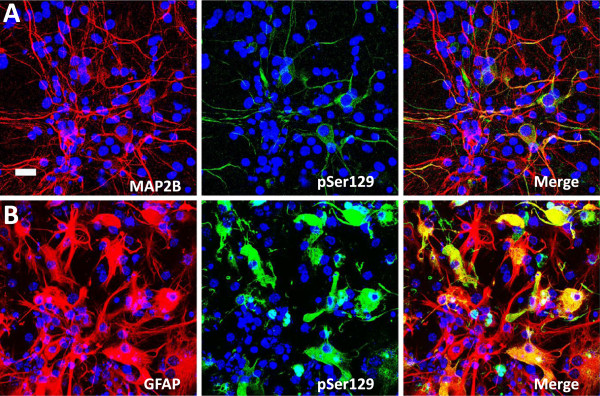
**αS aggregate formation in neurons and astrocytes.** (**A**) αS pSer129 positive aggregates (green) induced by treatment with extracellular αS fibrils in cultures with rAAV2/1 human αS expression can occur in neurons as shown by double immunofluorescence with the neuronal marker MAP2B (red). (**B**) αS pSer129 positive aggregates (green) induced by treatment with extracellular αS fibrils in cultures with rAAV2/1 human αS expression can occur in astrocytes as shown by double immunofluorescence with the astrocyte marker GFAP (red). Cultures were counter stained with DAPI (blue). Bar scale = 25 μm.

To assess which types of cells were prone to display αS aggregate formation; we performed double immuno-fluorescence with the neuronal specific marker MAP2B and the astrocyte specific marker GFAP (Figure 4). Inclusion formation was readily observed in both cell types. In many experiments 4 days post-seeding, more than 50% of both SNL-4+ MAP2+ neurons and GFAP+ SNL-4+ astrocytes demonstrated pSer129+ aggregates using the rAAV2/1 wild-type αS and wild-type fibril combination. By 8 days post-seeding, nearly all cells that were transduced with the rAAV2/1 αS and were SNL-4+ showed pSer129+ inclusions. As the rAAV2/1 vectors efficiently transduce greater than 50% of astrocytes and neurons in culture, these studies reveal that this system shows remarkable efficiency of inclusion formation. Although αS is predominantly found at higher endogenous levels in neurons [39,42-44], by using rAAV driven expression, astrocytes are also as vulnerable to αS aggregate formation.

**Figure 5:**
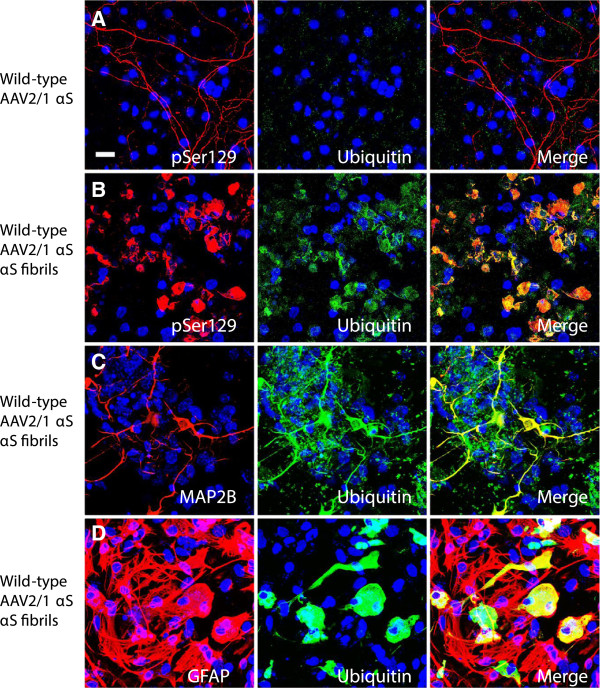
**αS inclusions in the mixed neuronal-glial cultures co-localize with ubiquitin.** (**A**) In cultures treated with rAAV2-1 human wild-type αS, pSer129 (red) neuritic background staining does not co-localize with endogenous ubiquitin (green). (**B**) In cultures treated with rAAV2/1 human wild-type αS and exogenous human wild-type αS fibrils, pSer129 (red) hyperphosphorylated αS inclusions in these cultures are ubiquitinated (green). This ubiquitin staining profile (green) co-localizes with the neuronal marker MAP2B (red, **C**) and the astrocyte marker GFAP (red, **D**). Cultures were counter stained with DAPI (blue). Bar scale = 25 μm.

**Figure 6:**
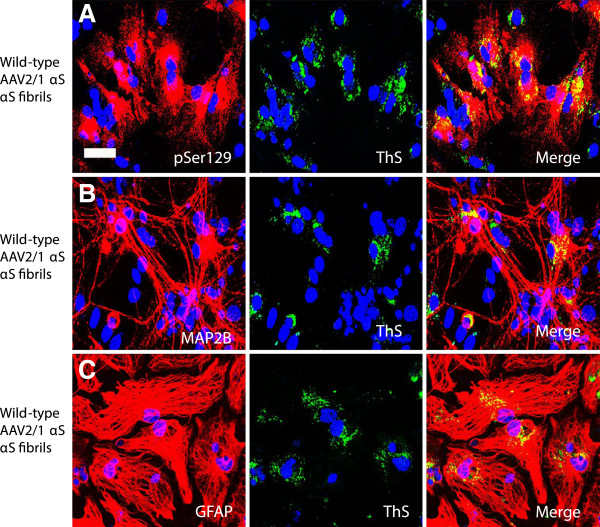
**αS inclusions induced in mixed neuronal-glial cultures contain Thioflavin**- **S positive structures.** (**A**) In cultures treated with rAAV2/1 human wild-type αS and human wild-type αS fibrils, pSer129 (red) hyperphosphorylated αS inclusions were also ThS positive (green). This ThS staining profile (green) co-localizes with the neuronal marker MAP2B (red, **B**) and the astrocyte marker GFAP (red, **C**). Cultures were counter stained with DAPI (blue). Bar scale = 50 μm.

αS aggregates formed in our cellular system are similar to those observed in human brains. The inclusions are partially ubiquitinated and are Thioflavin-S (ThS) positive (Figures 5 and 6). In cultures treated with rAAV2/1 human wild-type αS, the neuritic staining profile seen with pSer129 does not overlap with endogenous ubiquitin (Figure 5A). The overexpression of human wild-type αS (via rAAV2/1) plus exogenous human αS fibrils produced pSer129+ αS aggregates that co-localize with ubiquitin (Figure 5B). Co-localization of ubiquitin staining with pSer129+ αS aggregates is seen both in neurons (Figure 5C) and astrocytes (Figure 5D). Similarly, pSer129+ aggregates show co-localization with ThS (Figure 6A). The ThS staining profile is seen in both neurons (Figure 6B) and astrocytes (Figure 6C). However, it appears that ThS only stains a fraction of the pSer129 immunoreactive inclusions, suggesting that even within individual cells the structure of the aggregate is not homogenous.

### Morphological differences in αS pathology seen in primary mixed neuronal-glial cultures

**Figure 7:**
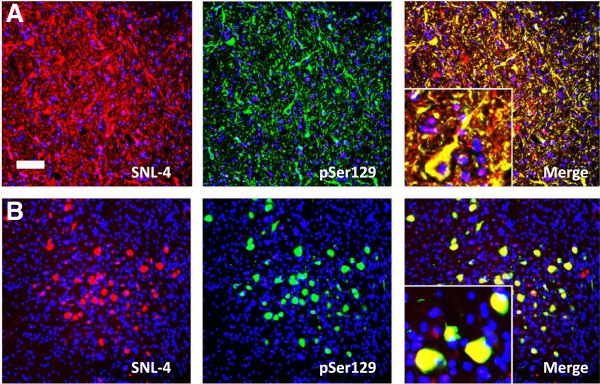
**Distinct morphological properties of αS inclusions in M83 (A53T) and M47 (E46K) human αS transgenic mice.** (**A**) Inclusions in M83 transgenic mice stained with αS antibody SNL-4 (red) or pSer129 (green) show a “flame-like” morphology. (**B**) Inclusions in M47 transgenic mice stained with αS antibody SNL-4 (red) or pSer129 (green) show a “round-like” compact morphology. Sections were counter stained with DAPI. Bar scale = 25 μm.

**Figure 8:**
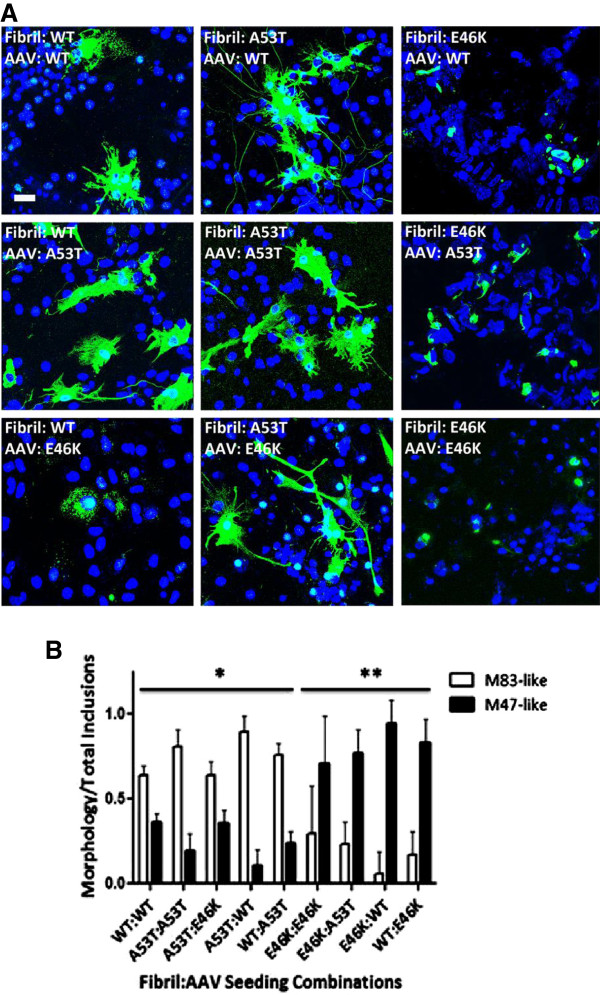
**αS mutant specific and dominant morphological induction of αS aggregates.** (**A**) Immunofluorescence analysis of morphological differences in αS aggregates is predominantly determined by the type of extracellular αS fibril challenge. Cultures were transduced with rAAV2/1 expressing wild-type, A53T, or E46K αS as indicated in each respective panel and described in Materials and Methods. Cultures were then treated with preformed fibrils comprised of wild-type, A53T, or E46K αS, as indicated in each respective panel. At one day post-seeding (and four days post-seeding for wild-type-wild-type), cultures were fixed and stained with pSer129 antibody (green) to assess for differences in morphology. Cells were counterstained with DAPI (blue). Bar scale = 25 μm. (**B**) Quantification of morphological differences for αS aggregates reveals statistical differences in inclusion morphology among seeding combinations. One-way ANOVA with Bonferroni correction for comparison of all groups to each other for flame-like M83 pathology reveals significant differences in inclusion morphology between all A53T fibrillar αS-seeded and E46K fibrillar αS-seeded (**) cultures, but not with WT and A53T combinations (*). Wild-type-wild-type combination (*) was more likely to result in M83-like inclusion morphology; however, when wild-type fibrils were used to seed either intracellularly expressed mutant proteins, the mutant protein predominantly determined inclusion morphology further indicating the dominant effect of the mutant proteins. p < 0.01; n = 6 fields/seeding combination. Values are means ± SD.

**Figure 9:**
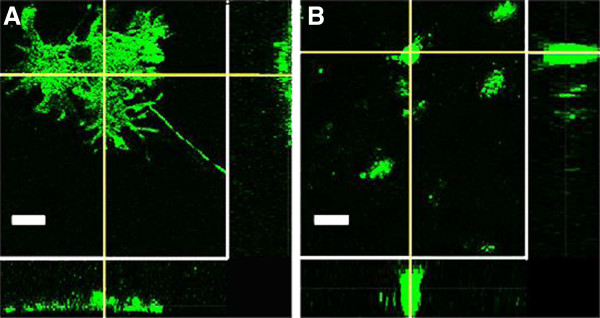
**Three-dimensional differences in morphology of αS inclusions.** Confocal Z slice analysis midway through the pSer129+ αS aggregates (green) show that (**A**) M83-like inclusions derived from rAAV2/1-mediated overexpression of human A53T αS and addition of A53T-derived human αS fibrils have a diffuse distribution throughout the cell body. In contrast, (**B**) M47-like inclusions derived from rAA2/1-mediated overexpression of human E46K αS and addition of E46K-derived human αS fibrils show rounded, punctate staining. Bar scale = 10 μm.

Transgenic mouse models for human A53T (line M83) and E46K (line M47) αS have been developed and characterized [14,15]. Both transgenic models express similar levels of human αS under the control of mouse prion protein promoter and develop similar age-dependent motor phenotypes associated with similar widespread distribution of neuronal αS inclusions. However, the αS pathology in the M83 transgenic model predominantly demonstrates a flame-like somatodendritic and neuritic profile; whereas, those seen in the M47 transgenic model have a rounded and compact profile (Figure 7). We therefore studied the effects that the A53T and E46K mutations would have on the morphology of αS pathology, and whether these differences were due predominantly to the type of protein being overexpressed, the type of fibril seeding pathology, or both. As seen in Figure 8A, wild-type, A53T, and E46K αS fibrils are able to cross-seed αS pathology. Regardless of the protein being overexpressed, seeding with A53T fibrils overall induced flame-like somatodendritic inclusions that we will refer to as M83-like pathology; whereas, seeding with E46K fibrils induced more rounded and compact inclusions that we will refer to as M47-like pathology (Figures 8 and 9). Blinded quantification also showed that the morphology of the inclusions was heavily dependent upon the type of fibril used to seed the culture and that the ratios of M83 to M47-like pathologies were significantly different (all p < 0.01). As stated above, the rAAV2-1 wild-type αS/ wild-type αS fibril combination showed slower aggregation kinetics relative to combinations that included a mutant protein, but it resulted mainly in M83-like pathology; however, when wild-type was used to seed A53T or E46K αs, the predominant morphology depended upon the protein being expressed, suggesting that wild-type αS seeds are more mutable or heterogeneous (Figure 8B).

### Passaging of αS aggregates via cell lysates in astrocyte cultures

**Figure 10:**
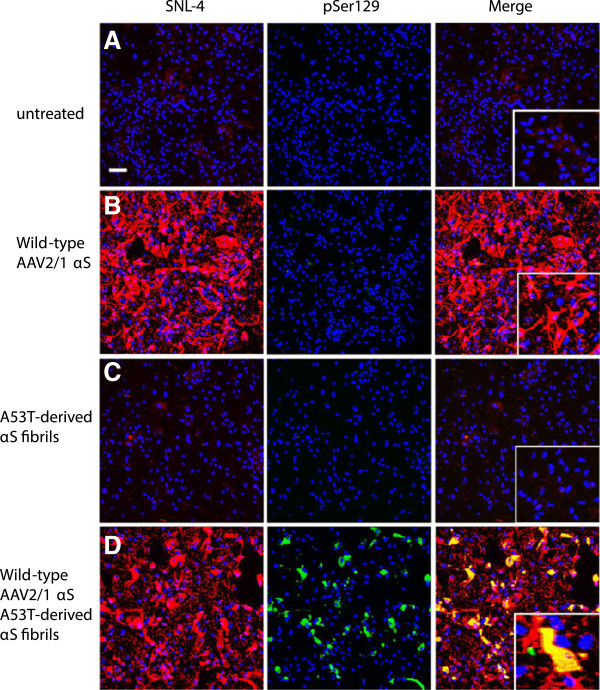
**αS seeded inclusion formation in astrocyte cultures.** Double immunofluorescence with SNL-4 (red) and pSer129 (green): (**A**) In untreated astrocyte cultures, there is very limited endogenous αS expression (SNL-4, red) and no hyperphosphorylated αS (pSer129, green). (**B**) rAAV2/1-mediated overexpression of human wild-type αS increases both the cell body and processes staining intensity for αS in the absence of hyperphosphorylated αS. (**C**) Addition of human A53T-derived αS fibrils alone showed similar endogenous expression levels of αS compared to the blank control along with the absence of hyperphosphorylated αS. (**D**) Both rAAV2/1-mediated overexpression of human wild-type αS and the addition of human A53T derived αS fibrils resulted in pSer129 immunoreactive inclusions in both the cell body and processes that overlap with SNL-4. Cultures were counterstained with DAPI (blue). Bar scale = 100 μm; insets = 25 μm.

Our studies showed that αS aggregate formation can be induced in both neurons and astrocytes (Figure 4). Primary astrocyte cultures have little to no endogenous αS (SNL-4, Figure 10A) and also lack the background neuritic staining of pSer129 (Figure 10A-D). As with the primary mixed neuronal-glial cultures, the formation of αS aggregates requires both the overexpression of αS (via rAAV2/1) and addition of exogenous fibrillar αS (Figure 10D). For passaging studies, cultures were treated with rAAV2/1 human wild-type αS and A53T-derived human αS fibrils.

**Figure 11:**
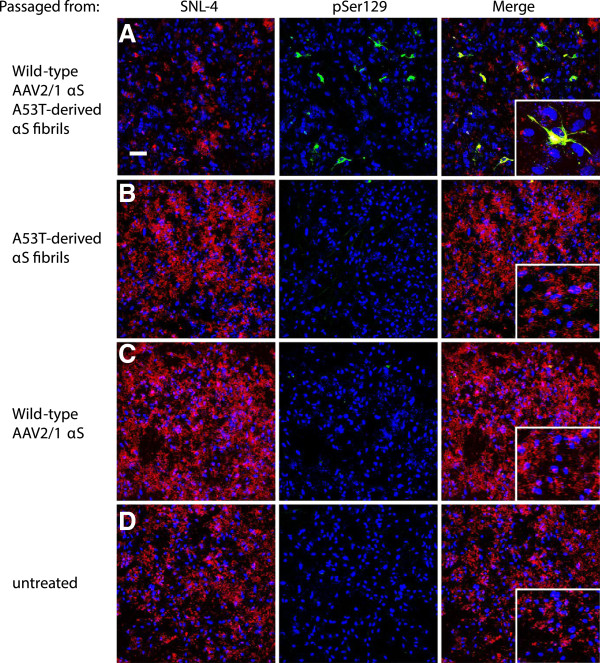
**αS species capable of inducting intracellular αS aggregates can be passaged from cell lysates.** (**A**) Astrocyte cultures with rAAV2/1-mediated overexpression of human wild-type αS treated with cell lysate from astrocyte cultures containing αS aggregates via overexpression of human wild-type αS and addition of human A53T- derived αS fibrils. At five days post-seeding, this resulted in pSer129 (green) aggregates in both the cell body and processes, which overlapped with SNL-4 (red). This was compared to astrocyte cultures with rAAV2/1-mediated overexpression of human wild-type αS and treated with cell lysates from astrocyte cultures treated with human A53T-derived αS fibrils (**B**), rAAV2/1-mediated overexpression of human wild-type αS (**C**), or untreated cultures (**D**). In the latter 3 conditions, at 5 days post-seeding there was uniformly distributed overexpression of αS in the absence of hyperphosphorylated αS aggregates (**B,C,D**). Cultures were counterstained with DAPI (blue). Bar scale = 100 μm; insets = 25 μm.

Cell lysates prepared as described in Material and Methods from the seeded cultures were added to astrocyte cultures overexpressing human wild-type αS (via rAAV2/1). The cultures were left to incubate with the lysate for 5 days. Cultures exposed to aggregate-containing cell lysate from the overexpression of human wild-type αS and seeding with A53T-derived human fibrils, showed passaging of pSer129 positive aggregates in 2.9±0.7% of transduced cells (Figure 11A). In contrast, astrocyte cultures overexpressing human wild-type αS and seeded with cell lysate from cultures that were treated with either A53T-derived fibrils (Figure 11B), rAAV2/1 human wild-type αS (Figure 11C), or nothing (Figure 11D), did not show any pSer129 and SNL-4 positive aggregates.

## Discussion

αS inclusions are a hallmark pathology seen in PD and related disorders termed synucleinopathies. Many studies have indicated that aggregated forms of αS can lead to neuronal demise [45-48]. Therefore it is important to better understand the mechanisms involved in αS aggregation to develop tools for therapeutic intervention. Previous *in vitro* studies have shown that soluble αS can efficiently undergo a conformational change into an amyloidogenic, fibrillar form and it is well established that *in vitro* αS aggregation into amyloid is a nucleation dependent process and can be greatly induced by the addition of a “seed” or “nucleus” of pre-aggregated αS [45-47,49,50].

Cellular studies have shown that the entry of a small amount of preformed αS fibrils into immortalized cell lines using reagents that promote the entry of these seeds across the plasma membrane can very efficiently induce the formation of large intracellular amyloid inclusions [32,33]. In another recent study, it was reported that the simple addition of extracellular αS fibrils to primary neurons can also induce the formation of intracellular αS inclusions [51]. However, in our primary neuronal-glial culture system, the addition of only fibrillar αS resulted in nuclear pSer129 immunoreactivity with some staining of the processes that did not co-localize with a marker for endogenous αS (SNL-4), and this staining was still present in cultures from αS null mice [52] indicating that it is primarily due to cross-reactivity. Although it is possible that some of the pSer129 immunostaining seen in neuritic processes of the seeded cultures does represent bonafide inclusion formation, it is imperative that other markers of αS inclusion pathology show co-localization with the neuritic pSer129 staining to distinguish inclusion formation from non-specific pSer129 staining. Indeed, the primary reliance on pSer129 immunostaining in both previous neuronal culture studies and *in vivo* may confound the interpretation of those studies. Overexpression of αS via rAAV2/1 plus the addition of fibrillar αS were both required for the rapid and efficient formation of αS pathology in our mixed primary neuronal-glial cultures. These aggregates are composed of endogenous αS, and share the biochemical properties of LBs and (Lewy neurites) LNs of being Ser129 hyperphosphorylated, ubiquitinated, Thioflavin-S positive, and Triton X-100 insoluble. They occur in both neurons and astrocytes in a high percentage of both cell types. Although LB and LN pathology in brain tissue samples from PD patients is predominantly seen in neurons, αS inclusions can occasionally also be within astrocytes along with a massive astrogliosis surrounding dying neurons in the SNpc of PD patients [53]. Using a mixed culture model where pathology is seen in both cell types allows for further study of the interplay that may be occurring during the pathogenesis of PD. Although subtle differences in experimental paradigms may well-explain the differences between our study and the Volpicelli-Daley et al. study where seeded inclusion formation was reported in primary neuronal cultures not overexpressing αS [51], this discrepancy will require further experimental investigation.

Some post-mortem studies of the distribution of αS pathology in the brains of PD patients and the induction of αS pathology in transplanted neurons in the brains of some PD patients have suggested that αS pathology may spread from peripheral nerves to the CNS and from the CNS onto grafted neurons [22-26], but different concepts have been proposed to explain the mechanism of pathology propagation. These have included: chronic generalized neuroinflammation, which may promote the up regulation and subsequent aggregation of αS; oxidative stress triggered by chronic excitotoxicity, which causes post-translational modifications of αS, such as nitration, that makes the protein more aggregate prone; loss of homeostasis from chronic cellular stress, which may lead to the failure of molecular chaperones and other machinery to effectively control the level of misfolded αS; and finally a “prion-like” spread where misfolded αS is transferred among cells and recruits endogenous αS into pathologic inclusions [54].

Further support for the notion that αS pathology may spread in the CNS by intercellular release and re-uptake of amyloidogenic αS are the recent reports showing that intracerebral injection of pre-formed recombinant αS fibrils or extracts from sick A53T human αS transgenic mice (line M83) into younger healthy M83 transgenic mice induces αS pathology and motor disease [27,55]. Injection of pre-formed recombinant αS fibrils into wild-type mice was also recently shown to induce brain αS aggregation that appears to spread from the site of injection [28]. These findings suggest that amyloidogenic αS species can initiate and perhaps lead to transmission of αS pathology, but the possibility that the other mechanisms mentioned above may also be involved cannot be excluded.

To further investigate the permissive conformational templating of αS aggregation, we took advantage of the unique properties of disease-causing mutants A53T and E46K, which also have unique effects on aggregate formation. Both mutations are found in the amino terminus of the protein, proximal to the central hydrophobic stretch (NAC region), which is required for fibrillization of αS and they accelerate the fibrillization of αS with the A53T mutation having an increased propensity over the E46K mutation [18,31,56,57]. The increased aggregation of these two mutants of αS is due to alterations in the peptide structural order of αS, which influences monomer folding and may make them more susceptible to aggregation [19,20]. In addition, *in vitro* polymerized E46K αS fibrils show district structural and biochemical features compared to wild-type and A53T αS fibrils [17-19,31] and these differences are most likely directly responsible for the different observed morphological conformations of the αS inclusions in cultured cells and transgenic mice. Our findings that primary cultures expressing wild-type, E46K, or A53T αS challenged to A53T-derived αS fibrils mainly resulted in aggregates with a flame-like profile, while similar treatment with E46K-derived αS fibrils primarily induced the formation of round, compact inclusions demonstrate the conformational templating of αS aggregation *in vivo*. It is not clear if the differences in inclusion morphologies result in any significant altered pathophysiological outcomes or if they simply reflect different structure variants. Mice that express E46K or A53T human αS develop pathological inclusions with these distinct morphological features, but both types of inclusions are associated with similar severe motor phenotype resulting in death [14,15].

These data, along with other evidence that αS pathology may to be transmissible [22-24,27,28,58], indicates that αS aggregation may be able to spread intercellularly by conformational templating of amyloid formation akin to a strain-specific prion-like mechanism that has been documented for other prionoid diseases [29,30]. One difference between our model and typical prion strain-specific properties [29,30] is that we studied this phenomenon for αS using disease-causing mutants, while the same type of process can occur with wild-type prion proteins. Future studies will investigate whether unique post-translational modifications of wild-type αS can also result in “strain-like” conformational templating. Because both intrinsic perturbations of proteostasis and inflammatory stimuli can induce αS inclusion formation it is currently challenging to definitively distinguish between true seeding of pathology and other factors that may contribute to inclusion formation *in vivo*[54]. Based on the morphological seeded templating described here, it should be possible to distinguish between true exogenous seeding and other mechanisms of pathology induction *in vivo*.

We also show that αS pathology can be induced in multiple CNS cell types, allowing for studies of how the interplay between these cells may contribute to disease. Because of its efficiency, future studies examining both the prion-like spread and its effect on cellular function can be facilely carried out using this culture model. Further work to provide more support for the “prion-like” spread can be done using this model. One of the characteristics of a “prion-like” spread is that pathology can be continuously passaged from an infected to naïve host and that different prion strains maintain their pathological characteristics [59]. Although we show that αS aggregation can be passaged from cell lysates derived from astrocytes cultures seeded with A53T αS fibrils, we are currently expanding our studies to further investigate this phenomenon. These studies will help us to elucidate the mechanisms by which pathology spreads not only in PD but also potentially in other neurodegenerative disorders and help to study a means for therapeutic intervention.

## Conclusion

We report on a novel mixed primary neuronal-glial culture system that can be used as a robust model of αS aggregate formation. A major advantage of this system is that we can manipulate both the αS protein expressed and the type of extracellular seeds added. Using this experimental system and disease-causing αS mutants, we show that the addition of extracellular preformed fibrils can induce conformational templating of intracellular aggregates and that the nature of mutant fibrils has a dominant effect on inclusion formation. These studies provide further credence to the notion that αS pathology may be able to spread via conformational dependent self-templating (“prion-like”) mechanisms at least in terms of intercellular transmission and induction of change in protein conformation by direct protein interaction. If such conformational templating occurs *in vivo*, then distinct inclusion morphology induced by distinct αS may be one surrogate that can be used to distinguish between seeded templating and other mechanisms of inducing αS pathology *in vivo*.

## Materials and methods

### Mixed neuronal-glial primary cultures

All procedures were performed according to the NIH Guide for the Care and Use of Experimental Animals and were approved by the University of Florida Institutional Animal Care and Use Committee. *SNCA* null mice [52] were obtained from The Jackson Laboratory (Bar Harbor, MA). Primary cultures were prepared from P0 C3HBL/6 mouse brains (Harlan Labs). Cerebral cortices were dissected from P0 mouse brains and were dissociated in 2 mg/mL papain (Worthington) and 50 μg/mL DNAase I (Sigma) in sterile Hank’s Balanced Salt Solution (HBSS, Life Technologies) at 37°C for 20 minutes. They were then washed three times in sterile HBSS to inactivate the papain and switched to 5% fetal bovine serum (HyClone) in Neurobasal-A growth media (Gibco), which includes 0.5 mM L-glutamine (Gibco), 0.5 mM GlutaMax (Life Technologies), 0.01% antibiotic-antimycotic (Gibco), and 0.02% SM1 supplement (Stemcell). The tissue mixture was then triturated three times using a 5 mL pipette followed by a Pasteur pipette, and strained through a 70 μm cell strainer. The cell mixture was then centrifuged at 200 g for 3 minutes, and re-suspended in fresh Neurobasal-A media. They were then plated onto poly-D lysine coated chamber slides (Life Technologies) or dishes at around 100,000-200,000 cells/cm^2^. Cells were maintained in the Neurobasal-A growth media mentioned above without fetal bovine serum at 37°C in a humidified 5% CO_2_ chamber.

### rAAV2/1 preparation and expression

rAAV serotype 2/1 expressing human wild-type, A53T or E46K αS under the control of the CMV early enhancer/chicken β actin (CAG) promoter, were generated as described previously [60]. At 5 days *in vitro* (DIV), each virus was added to cultures at a final concentration of 10^11^ genome copies/mL. We have determined that at 12DIV, ~40%+ of cells in our cultures are transduced by rAAV2-1.

### Preparation of recombinant αS fibrils

Recombinant, human β-synuclein and 21–140, wild-type, A53T, E46K, and ∆71-82 αS proteins were expressed and purified as described previously [31,32,34,41]. For amyloid assembly 21–140, wild-type, A53T, and E46K αS proteins (5 mg/mL) were incubated in sterile phosphate-buffered saline (PBS; Life Technologies, Carlsbad, CA, USA) at 37°C with continuous shaking at 1050 rpm (Thermomixer R, Eppendorf, Westbury, NY, USA) and fibril formation was monitored by turbidity and K114 fluorometry [32]. Fibrils were diluted to 1 mg/mL in sterile PBS and sonicated for 2 hours, which results in fragmentation into smaller fibrils of varying lengths [32,33]. Cultures were treated with 1 μM of fibril mix at 8 DIV.

Fibrils pre-labeled with Thioflavin-S (ThS) were only used where indicated. A 1 mg/mL fibril mix was incubated in 0.05% ThS for 1 hour then spun at 16,000 g for 5 minutes, and washed with PBS three to five times and sonicated for 2 hours. Fibril mix was then added to cultures at a final concentration of 1 μM.

### Biochemical cellular fractionation and western blotting analysis

Samples for biochemical analysis were harvested at 4 days post-seeding. Cultures were washed in PBS and scraped in 1% Tx-100 TBS (50 mM Tris, 150 mM NaCl, pH 7.4) with protease and phosphatase inhibitors and placed on ice for 10 min. Lysates were then centrifuged at 100,000 g for 20 min at 4°C. Supernatants were removed (Triton-soluble fraction), and the pellet was washed with the TBS buffer and re-centrifuged. The remaining pellet was then resuspended in 2% SDS, sonicated and heated to 100°C for 10 min (Triton-insoluble fraction). 2% SDS was added to the Triton-soluble fraction that was heated to 100°C. Equal amounts of protein were resolved by SDS-PAGE on 15% polyacrylamide gels, followed by electrophoretic transfer onto nitrocellulose membranes. Membranes were blocked in TBS with 5% dry milk, and incubated overnight with Syn211, a mouse monoclonal antibody specific for amino acids 121–125 in human αS [61], in TBS/5% dry milk, or pSer129, a mouse monoclonal antibody specific for αS phosphorylated at Ser129 [35], in TBS/5% bovine serum albumin (BSA). A total anti-actin antibody (clone C4) (Millipore, Billerica, MA) was used as a loading control. Each incubation was followed by goat anti-mouse conjugated horseradish peroxidase (HRP) (Amersham Biosciences, Piscataway, NJ). Protein bands were detected using chemiluminescent reagent (NEN, Boston, MA) and a FluorChem E and M Imager (Proteinsimple, San Jose, California).

### Immunofluorescence microscopy analysis

Samples for immunofluorescence analysis were taken at 4 days post-seeding (and at 1 day post-seeding where indicated). For double immunofluorescence analysis, cells were fixed with 4% paraformaldehyde/PBS. Following PBS washes, cells were blocked with 5% goat serum/PBS/0.3% Triton X-100 for 1 hour. Cultures were incubated in primary antibodies: pSer129 (1:500) and SNL-4 (1:500), a rabbit polyclonal antibody raised against amino acid sequence 2–12 of human αS, but that also reacts with murine αS [61]. Other primary antibodies include rabbit polyclonal anti-MAP2B (1:100), a neuronal marker (Millipore); rabbit polyclonal anti-GFAP (1:1000), an astrocyte marker (Dako); and rabbit polyclonal anti-ubiquitin (1:1000) (Abcam). This was followed by incubation with Alexa-fluor 488 and 594 conjugated secondary antibodies (1:1000) (Invitrogen). Nuclei were counterstained with 4′,6-diamidino-2-phenylindole (DAPI; Invitrogen), and coverslips were mounted using Fluoromount-G (Southern Biotech, Birmingham, AL). Thioflavin-S (Sigma-Aldrich) immunostaining was performed after secondary antibody incubation at a concentration of 0.05% followed by three washes in 70% ethanol and three washes in water. Images were captured on a Leica TCS SP2 AOBS Spectral Confocal Scanner mounted on a Leica DM IRE2 inverted fluorescent microscope. All images were captured with either 20× or 63× water immersion objectives as projection images from a Z-stack of <1.0 μm per plane.

### Astrocyte cultures

All procedures were performed according to the NIH Guide for the Care and Use of Experimental Animals and were approved by the University of Florida Institutional Animal Care and Use Committee. Astrocyte cultures were prepared from P2 C3HBL/6 mouse brains (Harlan Labs). Cerebral cortices were dissected from P2 mouse brains and were dissociated in 2 mg/mL papain (Worthington) and 50 mg/mL DNAase I (Sigma) in sterile Hank’s Balanced Salt Solution (HBSS, Life Technologies) at 37°C for 20 minutes. They were then washed three times in sterile HBSS to inactivate the papain and switched to Dulbecco’s Modified Eagle Medium (Life Technologies) with 10% fetal bovine serum (Life Technologies) and 100 U/mL Penicillin and 100 μg/mL Streptomycin (Life Technologies). The tissue mixture was then triturated three times using a 5 mL pipette followed by a Pasteur pipette and strained through a 70 μm cell strainer. The cell mixture was then centrifuged at 200 g for 3 minutes, and re-suspended in fresh DMEM media. They were then plated in chamber slides, 10 cm culture dishes, or T-75 flasks at 75,000 to 200,000 cells/cm^2^. Cells were maintained in the DMEM media mentioned above at 37°C in a humidified 5% CO_2_ chamber. After two days, flasks were shaken for 30 seconds and supplied with fresh media. Upon reaching confluency, flasks were split using TrypLE Express (Life Technologies) for two generations per original culture.

### Cell lysate passaging in astrocyte cultures

Since induction of αS aggregates was seen in both neurons and astrocytes in mixed cultures, astrocyte cultures were used because they can be split which facilitated the passaging experiments. The first generation astrocyte cultures were split into 10 cm culture dishes at 75,000 cells/cm^2^. rAAV 2/1 expressing human wild-type αS, as described above, was added at 3 days following plating at a final concentration of 10^11^ genome copies/mL. At 5 days following plating, the media was changed and cultures were treated with 1μM αS fibril mix for 5 days. Cultures were then washed three times in PBS and incubated in 0.25% Trypsin-EDTA (Life Technologies) for 10 minutes to inactivate residual extracellular αS fibrils. Cells were then collected in DMEM and spun at 200 g for 5 minutes. Pellets were rinsed with PBS and then frozen.

The second generation astrocyte cultures were again split and were transduced with rAAV 2/1 expressing human wild-type αS, as mentioned above, for two days. Then the cell pellets from the first generation cultures were thawed, re-suspended in 1 mL PBS, and lysated by sheer force with a 27 g needle. 100 μL of this lysate was then added to the second generation astrocyte cultures for 5 days.

### Quantitative analysis

Observations of morphological differences in αS were made by observer blinded, random field counting. Images were captured with an Olympus BX51 fluorescence microscope mounted with a DP71 digital camera (Olympus, Center Valley, PA) using 10× magnification and imaged were enlarged with Photoshop software and using a grid to allow for total field counting. For each seeding combination, 6 fields were counted. Criteria for inclusion morphology were set according to that seen in the M83 and M47 mouse models (see Results Figure 7). Briefly, either M83-like filling the somatodendritic compartment or M47-like with compact and rounded perinuclear inclusions. Results were expressed as the mean ratio of cells showing either type of morphology to the total number of inclusions per field ± SD. Comparisons for significance were made using one-way ANOVA with Bonferroni’s post-hoc test in GraphPad Prism software (San Diego, CA).

## Abbreviations

αS: α-Synuclein; LB: Lewy body; LN: Lewy neurite; PD: Parkinson’s disease; rAAV: recombinant adeno-associated virus.

## Competing interests

The authors declare that they have no competing interests.

## Authors’ contributions

ANS designed the study, performed culture experiments, analyzed the data, and drafted the manuscript. MAT performed culture experiments, and analyzed the data. CCD participated in the design of the study and performed culture experiments. PEC constructed the rAAV2/1 used and performed culture experiments. AMR helped to construct the rAAV2/1 used and performed culture experiments. JL participated in the design and coordination of the study. BIG and TEG participated in the design and coordination of the study, analyzed the data, and helped to draft the manuscript. All authors read and approved the final manuscript.
